# A study on the influence of internet addiction and online interpersonal influences on health-related quality of life in young Vietnamese

**DOI:** 10.1186/s12889-016-3983-z

**Published:** 2017-01-31

**Authors:** Bach Xuan Tran, Le Thi Huong, Nguyen Duc Hinh, Long Hoang Nguyen, Bao Nguyen Le, Vuong Minh Nong, Vu Thi Minh Thuc, Tran Dinh Tho, Carl Latkin, Melvyn WB Zhang, Roger CM Ho

**Affiliations:** 1 Institute for Preventive Medicine and Public Health, Hanoi Medical University, Hanoi, Vietnam; 2 Bloomberg School of Public Health, Johns Hopkins University, Baltimore, MD USA; 3 School of Medicine and Pharmacy, Vietnam National University, Hanoi, Vietnam; 4 Institute for Global Health Innovations, Duy Tan University, Da Nang, Vietnam; 5 Department of Hepatobiliary Surgery, Viet-Duc Hospital, Hanoi, Vietnam; 6 Biomedical Global Institute of Healthcare Research & Technology (BIGHEART), National University of Singapore, Singapore, Singapore; 7 Department of Psychological Medicine, Yong Loo Lin School of Medicine, National University of Singapore, Singapore, Singapore; 8 Department of Immunology and Allergy, National Otolaryngology Hospital, Hanoi, Vietnam

**Keywords:** Internet addiction, Interpersonal influences, Quality of life, Vietnam, Young people

## Abstract

**Background:**

Internet addiction (IA) is a common problem found in young Asians. This study aimed to study the influence of IA and online activities on health-related quality of life (HRQOL) in young Vietnamese. This study also compared the frequencies of anxiety, depression and other addiction of young Vietnamese with and without IA.

**Methods:**

This study recruited 566 young Vietnamese (56.7% female, 43.3% male) ranging from 15 to 25 years of age via the respondent-driven sampling technique. Chi-squared, *t*-test and analysis of variance were used to compare young Vietnamese with and without IA. Regression analyses were used to examine the association between internet usage characteristics and HRQOL.

**Results:**

Results from this cross-sectional study showed that 21.2% of participants suffered from IA. Online relationship demonstrated significantly higher influences on behaviors and lifestyles in participants with IA than those without IA. Participants with IA were more likely to have problems with self-care, difficulty in performing daily routine, suffer from pain and discomfort, anxiety and depression. Contrary to previous studies, we found that there were no differences in gender, sociodemographic, the number of participants with cigarette smoking, water-pipe smoking and alcohol dependence between the IA and non-IA groups. IA was significantly associated with poor HRQOL in young Vietnamese.

**Conclusion:**

IA is a common problem among young Vietnamese and the prevalence of IA is the highest as compared to other Asian countries. Our findings suggest that gender may not play a key role in IA. This can be an emerging trend when both genders have equal access to the internet. By studying the impact of IA on HRQOL, healthcare professionals can design effective intervention to alleviate the negative consequences of IA in Vietnam.

## Background

In the past 20 years, internet has become an integral part of our lives and an important tool for social interaction and communication [1]. Access to internet is affordable and there has been a rapid growth of users in developing countries. Excessive internet use has led to negative impact on the health of users [2].

A body of research suggests that problematic Internet use can be viewed as an addictive behavior [3, 4]. Signs and symptoms of Internet addiction (IA) include preoccupation, mood symptoms consistent with withdrawal, greater time spent (tolerance) and functional impairment or negative consequences due to excessive use. IA may include internet gaming and other forms of addictive internet usage which include excessive downloading, use of social networking sites and online shopping [5]. While internet is an integral part of our daily life, IA is increasingly more common among young people and has become a pandemic worldwide [6]. For young people, poor social support and social isolation have been shown to result in IA [7]. Furthermore, IA may also have a negative impact on social skills, and interpersonal relationship [8]. Hence, it is important to assess the relationship between online interpersonal influences and IA because young people suffering from IA are often shy [9] and have low social skills [10]. The negative consequences of low social skills associated with IA remain unknown [2]. No studies to date have explored the relationship between IA and online interpersonal influences.

IA leads to negative consequences on mental health. A meta-analysis comprising 1641 patients suffering from IA and 11210 healthy controls found that IA was significantly associated with alcohol abuse, attention deficit and hyperactivity, depression, and anxiety [5]. IA may be associated with other forms of addiction including smoking and alcohol dependence [11, 12]. Andrews et al. (2002) found that peer influences contributed to substance use among young people [13]. Besides adverse psychological problems, IA also causes physical problems including back pain and strain injury [14]. If IA is not intervened upon early, it may lead to adverse effects on both physical and mental health in young people.

In 2013, the six-nation survey was conducted and compared the prevalence of IA among young Asians in China, Hong Kong, Japan, South Korea, Malaysia, and the Philippines [15]. IA was common among young people in these Asian countries and the prevalence of IA was highest in Philippines (21%). The reason for the high prevalence of IA among young Asians may be due to the fact that they often face the conflicts between collective culture [16] and individual identity formation [17]. Online activities allow young Asians to avoid awareness of their actual self and real-life problems [16]. Young Asians may engage in online activities such as online gaming to avoid conflict between the collective culture and their identity formation [16]. In China, problematic internet use was associated with psychosomatic symptoms and life dissatisfaction [14]. In Taiwan, the risk factors for IA were male gender, mental health comorbidity, and poor social support [18]. It is important to study IA among youth in other Asian countries because young people constitute majority of internet users and some of them exhibit addictive behaviors towards internet [18]. One important country which was not included in the 2013 six-nation survey was Vietnam.

The prevalence of IA in Vietnam is unknown. Son et al. (2012) found that young male Vietnamese who were addicted to multiplayer online role-playing game had higher scores on mental disorder scale [19]. Addiction to online game does not represent the whole spectrum of IA. Vietnam is one of the fastest-growing economies and the Kinh ethnic group constituted around 86% of the population. The situation of IA remains unknown in the the Kinh ethnic group which emphasizes on family bonding and spirituality which includes the practice of ancestor worship. In 2015, Vietnam had 44.4 million internet users and is projected to grow to 55.8 million internet users in 2018 [20]. Given the high broadband penetration rate in Vietnam, there is no doubt that IA is becoming increasingly problematic among young Vietnamese. IA is relatively less studied in Vietnam as compared to other Asian countries because the health care system focuses more on physical diseases [19]. Furthermore, there is lack of data for IA in young female Vietnamese.

In this study, we surveyed the prevalence of IA and health-related quality of life (HRQOL) via internet with a specific focus on young Vietnamese who are vulnerable to IA due to access to internet and computer literacy. The objective of this study was to investigate the association of IA, online interpersonal influences and HRQOL. First, we compared the differences between young Vietnamese with and without IA. Next, we investigated the association between online behaviors, HRQOL, physical and mental health problems. We hypothesized there were significant differences between young Vietnamese with and without IA in (i) socio-demographic characteristics; (ii) different domains of online interpersonal influences; (iii) the occurrence of physical and mental health problems; (iv) HRQOL and (v) occurrence of other forms of addiction. By identifying factors associated with poor HRQOL, this study aims to identify targets for future health interventions to improve HRQOL, physical and mental health of young Vietnamese in the era of internet and online culture.

## Methods

### Participants and Procedures

A cross-sectional study using web-based survey was conducted from August to October 2015 in Vietnam. The study was approved by the Institutional Review Board of the Hanoi Medical University. The inclusion criteria were: 1) Age from 15 to 25 years; 2) Currently living in Vietnam; 3) Agreement to participate in this study by providing the consent online. 4) Having a valid email account or account of social network sites to recruit other participants through the respondent-driven sampling (RDS) technique. The sample size was calculated by using the formula of Wejnert et al. [21] for RDS technique. With expected prevalence of youths being addicted to the Internet = 12.3% (according to a previous study in Vietnam [22]), Confident level = 95%; margin of error = 0.05 and design effect for RDS = 3, the minimum sample size was 498 youths. We add 15% to the sample size to compensate for people having incomplete answer. The final sample size was 573. After data collection, 566 youths were included into the data analysis phase.

The initial stage of recruitment focused on several core groups from various universities and high schools in Vietnam including the Hanoi Medical University, Vietnam National University, Hung Yen high school, and Phan Boi Chau high school. These groups were selected to reflect the diversity of study population by age, gender, and levels of education. These initial participants were more likely to know other young Vietnamese, who shared similar characteristics which made them eligible to meet the inclusion criteria. Based on the respondent-driven sampling technique, the initial participants were asked to recruit up to 5 other suitable participants through their social network.

### Measures

Before the initiation of data collection, a pilot study was conducted on 20 young participants with different age and gender. These participants assessed the online platform and provided recommendations to enhance its accessibility and usability. The web-based survey included following sub-scales:

1. Socio-demographic questions including age, gender, education, occupation, marital status, ethnicity and religion.
2. The HRQOL was measured by using the EuroQol - five dimensions - five levels (EQ-5D-5 L) and EuroQol -visual analogue scale (EQ-VAS). The EQ-5D-5 L includes five domains: Mobility, Self-care, Usual Activities, Pain/Discomfort and Anxiety/Depression with five levels of response: no problems, slight problems, moderate problems, severe problems, and extreme problems, giving 3125 health states with respective single indexes. The EQ-VAS allowed the respondents to rate their health status on a 20-cm vertical scale, with the endpoint ranging from 0 to 100 points, labeled from ‘the worst health which you can imagine’ to ‘the best health which you can imagine’.
3. The original form of Internet Addiction Test (IAT) was developed by Young et al. [23], comprising of 20 items with 5-point scale from 1 (“rarely”) to 5 (“always”) to measure various aspects of IA such as loss of control, time management and impairment in performance. The IAT has been used extensively in Asia [24]. In this study, we adapted the IAT (short form) which was validated by Pawlikowski et al. [25]. The short form consists of 12 items with good psychometric properties and assessing key features of IA based on diagnostic criteria [25]. The participant used a 5-point Likert scale to indicate their responses ranging from 1 (“rarely”) to 5 (“always”) and the scores ranged from 12 to 60 points. Higher scores suggest higher levels of IA. The cut-off score of 36 was used to identify participants with potential IA [26]. This questionnaire was translated into Vietnamese. To ensure the validity and reliability of this version, we applied the WHO’s guideline for translation and adaptation of instrument [27]. We involve two experts in English and Vietnamese to translate this instrument. Both of them were also experts in the field of medicine and psychology. We did forward translation, expert panel and back-translation as the recommendations from guideline. Then, we piloted the Vietnamese instrument with 10 youths and corrected any words or statements that could lead to misunderstandings. The Cronbach’s alpha of this instrument was 0.8667.
4. To measure the level of alcohol abuse, the Alcohol Use Disorders Identification Test-Consumption (AUDIT-C) questionnaire was used. The Vietnamese version of this scale was used and validated in previous studies [28, 29]. The AUDIT-C is commonly used by primary care physicians in order to screen for alcohol abuse [30]. The AUDIT-C consisted of three questions with scoring from 0 to 12 points, when higher scores indicate higher risk of alcohol dependence. If male respondents had score ≥ 4 and female respondents had score ≥ 3, they would be classified as potential cases of alcohol dependence [30].
5. We investigated the online interpersonal influences on participants including the frequency of communicating with online friends, self-perception on the effects of online relationship on behaviors, lifestyles and perception, visiting places recommended by online friends and engagement of activities recommended by online friends.
6. We collected other information including the time spent by each participant on Facebook, current status on cigarette smoking, and water-pipe (shisha) smoking.

### Statistical analysis

STATA software version 12.0 (Stata Corp. LP, College Station, United States of America) was used to analyze the data. *T*-test, Mann–Whitney test, Chi-squared test and Fisher’s exact test were used to explore the differences between respondents with and without IA. Multivariate linear regression was used to identify factors associated with poor HRQOL, pain/discomfort and anxiety/depression. In this study, we applied a stepwise forward model strategy which used the log-likelihood ratio test with p-value set at 0.1 to select variables for the regression model. A p-value of less than 0.05 was set as the level of statistical significance.

## Results

### Socio-demographic characteristics of participants

Table 1 summarizes the socio-demographic characteristics of participants. Using the IAT cut-off of 36, one hundred twenty out of 566 participants (21.2%) suffered from IA. The mean age of participants identified with IA was 21.8 years while the mean age of participants without IA was 21.4 years. Among the 120 participants with IA, the number of male participants was 52 (43.3%) and female participants were 68 (56.7%). For participants with and without IA, most of them had high school education and above, the Kinh ethnicity, cult of ancestor as religion and average economic status. There were no significant differences between participants with and without IA in mean age, gender, education attainment, ethnicity, religion, marital status, current living location and economic status (*P >* 0.05).

**Table 1:** Comparison of socio-demographic characteristics of participants with and without internet addiction

	Internet addiction						*p*
	Yes		No		Total		
	*n*	%	*n*	%	*N*	%	
Number of participants	120	21.2	446	78.8	566	100.0	
Mean age (SD)	21.8	(3.9)	21.4	(3.7)	21.5	3.8	0.32*
Gender							
Male	52	23.6	168	76.4	220	38.9	0.26**
Female	68	19.7	278	80.4	346	61.1	
Education attainment							
≤ High school	5	17.2	24	82.8	29	5.1	0.59**
> High school	115	21.4	422	78.6	537	94.9	
Ethnicity							
The Kinh ethnicity	116	21.5	424	78.5	540	95.4	0.46**
Other ethnicities	4	15.4	22	84.6	26	4.6	
Religion							
Cult of Ancestor	109	22.5	376	77.5	485	85.7	0.70**
Other religions	11	13.6	70	86.4	81	14.3	
Marital status							
Single	94	22.0	333	78.0	427	75.4	0.41**
Living with spouse/partner	26	18.7	113	81.3	139	24.6	
Current living location							
Renting a hostel	62	23.4	203	76.6	265	46.8	0.45***
Staying in dormitory	16	22.9	54	77.1	70	12.4	
Living with family	36	20.1	143	79.9	179	31.6	
Living with relatives	5	11.6	38	88.4	43	7.6	
Other liver arrangements	1	11.1	8	88.9	9	1.6	
Economic status of family							
High	1	7.7	12	92.3	13	2.3	0.09***
Average	99	20.2	392	79.8	491	86.8	
Low	18	32.7	37	67.3	55	9.7	
Very low	2	28.6	5	71.4	7	1.2	

*Student t-*test*; **Chi-*squared test*; ***Fisher’*s exact test*

### Forms of interpersonal influences from online relationship

Table 2 compares the various forms of interpersonal influences on lifestyles and social activities from online relationship on participants with and without IA. Online relationship demonstrated significantly higher influences on behaviors and lifestyles on participants with IA (12.0%) than those without IA (5.3%, *p <* 0.01). Participants with IA were significantly more likely to visit places (*p =* 0.02) and engage in activities (*p <* 0.01) recommended by their online friends. Furthermore, participants with IA spent significantly more time on social media such as Facebook per day (*p <* 0.001).

**Table 2:** Comparison of online interpersonal influences on lifestyles and social activities between participants with and without internet addiction

	Internet addiction						*p*
	Yes		No		Total		
	*N*	%	*n*	%	*n*	%	
Frequency of communicating with friends online							
Often	11	9.3	29	6.7	40	7.2	0.22*
Frequently	32	27.1	94	21.6	126	22.8	
Rarely or never	75	63.6	312	71.7	387	70.0	
Self- perception of the effects of online relationships on behaviors and lifestyles							
High influence	14	12.0	23	5.3	37	6.7	<0.01*
Normal influence	37	31.6	82	19.0	119	21.7	
Little influence or no influence	66	56.4	327	75.7	393	71.6	
Visit places recommended by online friends							
Often	19	16.4	47	10.8	66	12.0	0.02*
Frequently	65	56.0	211	48.5	276	50.1	
Rarely or never	32	27.6	177	40.7	209	37.9	
Engage in activities recommended by online friends							
Often	18	15.3	23	5.3	41	7.4	<0.01*
Frequently	59	50.0	217	49.7	276	49.7	
Rarely or never	41	34.8	197	45.1	238	42.9	
Time spent on social media	Mean	SD	Mean	SD	Mean	SD	
Time for using Facebook (hours/day)	3.84	3.38	3.23	7.00	3.56	7.42	<0.01**

*Chi-*squared test*; ***Mann–*Whitney test*

### Health problems and health-related quality of life

Table 3 compares the occurrence of health problems and HRQOL between participants with and without IA. As compared to the counterparts, participants with IA were significantly more likely to have problems with self-care (*p <* 0.01), difficulty in performing daily routines (*p =* 0.04), suffer from pain or discomfort (*p =* 0.03) and anxiety or depression (*p <* 0.01). Participants with IA obtained significantly lower scores in EQ-5D (*p <* 0.001) and EQ-5D VAS (*p <* 0.001).

**Table 3:** Comparison of the occurrence of physical and mental health problems and health-related quality of life between participants with and without internet addiction

	Internet addiction				*p*
	Yes		No		
	*N*	%	*n*	%	
Difficulty with mobility	28	23.3	79	17.7	0.16*
Difficulty with self-care	19	15.8	32	7.2	<0.01*
Difficulty with usual activities	36	30.0	94	21.1	0.04*
Having pain or discomfort	69	57.5	207	46.4	0.03*
Suffering from anxiety or depression	102	85.0	325	72.9	<0.01*
	Mean	SD	Mean	SD	
EQ-5D index	0.69	0.2	0.75	0.2	<0.01**
EQ-5D VAS	76.7	17.2	81.1	16.0	<0.01**

*Chi-*squared test*; **Student t-*test*

### Occurrence of other forms of addiction among participants

Table 4 compares the occurrence of other forms of addiction between participants with and without IA. There were no significant differences between the occurrence of cigarette smoking, water-pipe smoking and alcohol dependence between participants with and without IA (*p >* 0.05).

**Table 4:** Comparison the occurrence of other forms of addiction in all participants (*n =* 566)

	Internet addiction				*p*
	Yes		No		
	N	%	n	%	
Current cigarette smokers	12	10.0	43	9.9	0.96*
Current water-pipe (Shisha) smokers	5	4.4	21	4.9	0.81*
Current dependence on alcohol	38	31.7	110	25.2	0.15*

*Chi-*squared test*

### Regression analysis

Table 5 shows regression analysis to explore the unique contribution of the univariate correlates in exploring the HRQOL of all participants. IA (β = −4.23, 95% CI = −7.76 to – 0.7), alcohol dependence (β = −4.93, 95% CI = − 9.02 to – 0.84) and moderate levels of self- perception online interpersonal influences on behaviors and lifestyles (β = −3.94, 95% CI = − 7.48 to −0.40) were significantly associated with negative EQ-5D scores. Similarly, IA (β = −0.061; 95% CI = − 0.102 to – 0.019) was significantly associated with negative EQ-VAS scores. In contrast, low levels of self- perception of online interpersonal influences on behaviors and lifestyles were significantly associated with positive EQ-VAS scores (β = 0.077, 95% CI = 0.040 to 0.115).

**Table 5:** Multivariate linear regression analysis exploring the association between internet use behaviors, other forms of addiction and health-related quality of life in all participants (*N =* 566)

	EQ-5D index			EQ-VAS		
	β	95% CI		β	95% CI	
Internet addiction (Yes vs No)	−4.23*	−7.76	−0.70	−0.061*	−0.102	−0.019
Duration of Facebook use/day (hours)	−0.05	−0.27	0.16	−0.002	−0.004	0.001
Shisha smoking (Yes vs No)	−5.78	−13.10	1.54			
Alcohol dependence (Yes vs No)	−4.93*	−9.02	−0.84			
Talk and meet new online friends (vs Often)						
Rarely or never	1.85	−1.68	5.38			
Effects of online relationships on behaviors, lifestyles and perception (vs High influence)						
Moderate influence	−3.94*	−7.48	−0.40			
Low influence or no influence				0.077*	0.040	0.115
Visit place introduced by online friends (vs Often)						
Rarely or never	−2.88	−5.87	0.12	−0.030	−0.064	0.004

*p < *0.05*

## Discussion

The aim of this pioneering study was to understand the interaction between IA, online interpersonal influences and HRQOL among young Vietnamese. The hypotheses that there were significant differences between young Vietnamese with and without IA in different domains of online interpersonal influences, the occurrence of physical and mental health problems and HRQOL were confirmed. In contrast, the hypotheses that there were significant differences between young Vietnamese with and without IA in socio-demographic characteristics and occurrence of other forms of addiction were not confirmed.

In this study, the prevalence of IA was 21.2% and it was established by a validated questionnaire, the IAT which was able to capture essential features of IA [11]. Our prevalence rate is higher or similar than other Asian studies (the prevalence of IA in Philippines was 21% (Mak et al. 2014); Korea was 20% [31]; Taiwan was 17.9% [18]; Singapore was 17.1% [32], Hong Kong was 16.4% [15]; Malaysia was 14.1% [15]; South Korea was 9.7% [15] and Japan was 6.2% [15]). The prevalence of IA in Vietnam is higher than the prevalence of IA reported in China [15, 33]. The prevalence of IA was reported to vary widely from study to study [14]. The variations could be caused by differences in the assessment methods for IA, as well as national differences in the prevalence of IA due to underlying cultural and social differences [14]. There is a possibility that IA is an emerging problem and prevalence of IA has increased since 2009. It is of utmost importance for each country to conduct studies to measure the prevalence of IA at regular intervals.

Contrary to findings from previous Asian studies, there was no significant difference between the IA and non-IA groups in the proportion of gender although previous Asian studies reported that male gender was a risk factor for IA [14, 18]. Researchers further postulated that online games and pornography were the main reasons contributing to IA in young men. Our findings suggest that young women are equally vulnerable to IA. This observation could be due to the fact that young men and women tend to be equal in manyaspects of life including the access to internet. Further studies are required to monitor the gender differences in IA in other countries. Young Vietnamese with IA were not more likely to be cigarette smokers, water-pipe smokers and alcoholics as compared to their counterparts without IA. This can be explained by the fact that the Kinh ethnic group views smoking water-pipe as part of their cultural practices and not associated with IA.

With regard to the forms of interpersonal influences from online relationship, online relationship demonstrated significantly higher influences on behaviors and lifestyles in young Vietnamese with IA. This study also showed that young Vietnamese with IA were significantly more likely to visit places and engage in activities recommended by their online friends. These are interesting findings since no studies to date have explored online interpersonal influences on lifestyles and behaviors in young people suffering from IA. These findings serve as a reference and require further replication in other countries. This is an expected finding because young Vietnamese with IA are spending ever-increasing amounts of time on internet. Internet is the only medium for socialization because the lack of social support from family and non-online friends is the main causes of IA [7]. From the cognitive perspectives, people with IA require greater cognitive efforts to make decision [34]. As a result, they may prefer to seek advices from online peers to help them deciding on activities or visiting places. From the social perspectives, one explanation is that young people with IA feel that they are safer or more comfortable with online communications [2], especially among those who suffer from IA and loneliness [18]. As a result, young people with IA are more open to suggestions by their online friends. Not surprisingly, young Vietnamese with IA spent significantly more time on social media such as Facebook on a daily basis.

Young Vietnamese with IA were more likely to report the occurrence of having problems in self-care and usual activities, pain or discomfort, anxiety or depression. These results are in line with previous research that has shown associations between IA and minor mental health morbidity [11, 14, 18]. Our findings confirm that IA could impair psychological well-being of young Vietnamese. Cao et al. (2009) suggested that excessive internet use often lead to heightened psychological arousal and result in health problems [14]. Primary care physicians need to assess physical and mental health status of young people with IA in developing countries. In addition, with regards to the HRQOL, young Vietnamese with IA had significantly lower scores in the EQ-5D index and EQ-5D VAS. These findings correspond to previous reports on IA and dissatisfaction with life [14]. The current finding confirms the results of previous research which found that long duration of internet use leads to functioning impairments [18]. The regression analysis showed that IA and alcohol dependence contribute to poor HRQOL in young Vietnamese. This finding suggests that IA could be as harmful as alcoholism.

### Clinical implications

The present research findings are of importance for future research on IA in developing countries. Our results help to develop targets for the evidence-based interventions to tackle adverse effects of internet on young Vietnamese. First, the intervention program must focus on male and female patients suffering from IA as both genders are vulnerable to IA. Second, the intervention program must penetrate all socio-economic sectors in Vietnam as there were no socio-economic differences between young Vietnamese with and without IA. Third, interpersonal psychotherapy is useful to help young Vietnamese suffering from IA by reducing the online interpersonal influences on their behaviors and lifestyles. Social skill training and role play are equally important to improve off-line communication and relationship. Behavior therapy and activity scheduling will help young Vietnamese with IA to re-establish daily routines. Fourth, doctors should assess for physical health problems (e.g. back pain) and mental health problems (e.g. anxiety and depression) in young Vietnamese presenting with IA. Fifth, the health authority should spend resources to tackle IA because the negative impact of IA on HRQOL can be as serious as other forms of addiction.

### Limitations

This study has several limitations. First, the respondent-driven sampling technique has its own limitation. This sampling depends on the first participants who determine the subsequent sampling and researchers have little control over the sampling method. This process is non-random and leads to potential sampling bias. Nevertheless, the respondent-driven sampling technique has its own advantages. This technique allows researchers to reach hidden population or people with a specific condition such as IA. Second, this cross-sectional study using online survey did not allow cause inferences to be drawn and it is possible that poorer health leads to greater internet use. Third, due to constraint of the length of online survey, we could not measure factors including personality and assess off-line relationship.

## Conclusion

This study found that IA is a common problem in young Vietnamese and the prevalence of IA is among the highest as compared to other Asian countries. Both genders are at risk for IA. Our study has contributed to the understanding of important interactions between IA, online interpersonal influences and HRQOL in young Vietnamese. The findings help health professionals to design evidence-based intervention to tackle adverse online interpersonal influences associated with IA in young Vietnamese.

## Abbreviations

AUDIT-C: Alcohol use disorders identification Test-consumption
EQ-5D-5 L: EuroQol - five dimensions - five levels
EQ-VAS: EuroQol -visual analogue scale
HRQOL: Health-related quality of life
IA: Internet addiction
IAT: Internet addiction test
RDS: Respondent-driven sampling
